# HIV and AIDS in the state of Paraná, Brazil, 2007-2022: trends and spatiotemporal distribution

**DOI:** 10.1590/1980-549720240015

**Published:** 2024-04-19

**Authors:** Rafaela Marito Montanha, Thamy Barbara Gioia, Antônio Carlos Vieira Ramos, Natalia Marciano de Araujo Ferreira, Miguel Angel Fuentealba Torres, Rosângela Aparecida Pimenta, Gilselena Kerbauy, Ricardo Alexandre Arcêncio, Flávia Meneguetti Pieri

**Affiliations:** I Universidade Estadual de Londrina – Londrina (PR), Brazil; II Universidade Federal de Goiás – Samambaia (GO), Brazil.; III Universidade de São Paulo – Ribeirão Preto (SP), Brazil.; IV Universidad de los Andes – Santiago, Chile.

**Keywords:** HIV, AIDS, Time series studies, Spatial analysis, Incidence, HIV, AIDS, Estudos de séries temporais, Análise espacial, Incidência

## Abstract

**Objective:**

The aim of this study was to analyze the spatiotemporal evolution of the incidence rates of human immunodeficiency virus (HIV) and acquired immune deficiency syndrome (AIDS) in the state of Paraná, Brazil.

**Methods:**

An ecological study with an analytical component of time series analysis was conducted in the state of Paraná from 2007 to 2022. The data source was the Notifiable Diseases Information System. To study the trend, the Prais–Winsten generalized linear regression model was used by decomposing the time series, and for spatial analysis, the Moran's index was applied.

**Results:**

The total sample consisted of 50,676 HIV/AIDS records. The incidence rate showed an increasing trend, with an average growth of 2.14% [95% confidence interval – 95%CI 1.16–3.13] per month. From 2007 to 2014 and from 2015 to 2022, the average number of cases in the state was 105.64 and 159.20 per 100,000 inhabitants, respectively, with significant variation among municipalities. Spatial clusters of high risk persisted in the metropolitan region, the capital, and coastal areas, and a new cluster was observed in the northern region of the state.

**Conclusion:**

The incidence rates of HIV/AIDS showed an upward trend over time. The number of cases varied considerably in some municipalities, especially in the coastal region. Spatial analysis revealed geospatial patterns of high risk in the main metropolitan areas of Paraná: Curitiba (including the coastal area), Londrina, and Maringá, which share characteristics such as a high degree of urbanization and ongoing economic development.

## RESUMO

**Objetivo:**

Este estudo teve como objetivo analisar a evolução espaçotemporal das taxas de incidência do vírus da imunodeficiência humana (HIV) e da síndrome da imunodeficiência adquirida (AIDS) no estado do Paraná, Brasil.

**Métodos:**

Foi realizado um estudo ecológico com componente analítico de séries temporais e análise espacial no estado do Paraná, de 2007 a 2022. A fonte de dados foi o Sistema de Informação de Agravos de Notificação. Para estudar a tendência, foi utilizado o modelo de regressão linear generalizada de Prais-Winsten por meio da decomposição de séries temporais e, para a análise espacial, foi aplicado o Índice de Moran.

**Resultados:**

A amostra total foi composta de 50.676 registros de HIV/AIDS. A taxa de incidência apresentou tendência crescente, com crescimento médio de 2,14% (intervalo de confiança de 95% — IC95% 1,16–3,13) ao mês. Nos períodos de 2007 a 2014 e 2015 a 2022, a média de casos no estado foi de 105,64 e 159,20 a cada 100 mil habitantes, respectivamente, com importantes variações entre os municípios. Agrupamentos espaciais de alto risco permaneceram na região metropolitana à capital e litoral e um novo agrupamento foi observado à região norte do estado.

**Conclusão:**

As taxas de incidência do HIV/AIDS apresentaram tendência temporal crescente. O número de casos variou substancialmente em alguns municípios, principalmente naqueles localizados na região litorânea. A análise espacial revelou padrões geoespaciais de alto risco nas principais regiões metropolitanas do Paraná: Curitiba (abrange o litoral), Londrina e Maringá, as quais compartilham características como elevado grau de urbanização e constante desenvolvimento econômico.

## INTRODUCTION

In 2022, an estimated 39 million people worldwide were living with the human immunodeficiency virus (HIV), including 2.2 million in Latin America. Brazil accounted for nearly half (990,000) of the people living with HIV in the Latin American region and had the third highest incidence of HIV among adults in 2022 (0.39% per 1000 uninfected population), behind Chile and Uruguay^1^.

A study assessing the spatial distribution and temporal evolution of the epidemic in Brazil revealed a higher incidence of cases in the southern region over a 16-year period^2^. The state of Paraná, currently the most populous in the region^3^, accounted for 29% of HIV cases over a 15-year period, making it the second state with the highest number of cases in southern Brazil and the 22nd among the federal units^4^.

Surveillance of HIV infection and the acquired immune deficiency syndrome (AIDS) is the responsibility of the Unified Health System (Sistema Único de Saúde-SUS), which focuses on three aspects: viral infection, progression to AIDS, and mortality. These data are provided by the Notifiable Diseases Information System (Sistema de Informação de Agravos de Notificação-SINAN) and other information systems. Both HIV and AIDS were included in the national list of notifiable diseases, with AIDS being included since 1986 and HIV infection since 2014^4^. This change was aimed at expanding the scope of diagnosis in order to improve prevention and control strategies^5^.

One of the main strategies for situational and epidemiological diagnosis in the context of HIV/AIDS relates to the use of spatial and temporal analysis tools, such as geoprocessing and time series, which contribute to the identification and understanding of disease transmission dynamics, providing evidence for prioritizing areas or zones for control and intervention activities^6^.

In a literature review conducted for this research, no studies were found that estimated the incidence of HIV/AIDS considering both spatial and temporal dimensions at the state level in Paraná. Therefore, this is the first study to analyze the disease in the country using these tools, contributing to the development of specific strategies and the creation and implementation of public policies aimed at addressing this health condition.

In this perspective, this study aimed to analyze the spatiotemporal evolution of the incidence rates of HIV and AIDS in the state of Paraná, Brazil, both in space and time, for the period from 2007 to 2022.

## METHODS

A mixed ecological study of temporal and spatial analysis was conducted^7^. The state of Paraná is in the southern region of Brazil and comprises 399 municipalities, with an estimated population of 11,835,379 inhabitants in 2022^8,9^.

Regarding the Health Care Network (Rede de Atenção à Saúde [RAS] in Portuguese), it comprises 177 establishments dedicated to the care of people living with HIV/AIDS^10^. This network is divided into four health macroregions, which, in turn, are decentralized into 22 health regions^11^.

Data from the Brazilian HIV/AIDS national database, reported in the Notifiable Diseases Information System (*Sistema de Informação de Agravos de Notificação*-SINAN) from 2007 to 2022, was used. The State Department of Health (*Secretaria de Estado da Saúde*-SESA) provided the data after receiving authorization from the Research Ethics Committee in September 2023.

All cases of HIV and AIDS reported in SINAN for individuals aged 13 years or older, residing in municipalities within the state of Paraná during the study period, were included. The case definition was based on the internal field contained in SINAN: case definition criteria (HIV+, Rio/Caracas, and Centers for Disease Control and Prevention (CDC) adapted for AIDS cases). Laboratory evidence of HIV/AIDS infection in adults in Brazil is considered for those aged 13 years or older. Cases without a recorded municipality or not belonging to the state had the case definition criteria discarded, and duplicate records were excluded.

**Figure 1:**
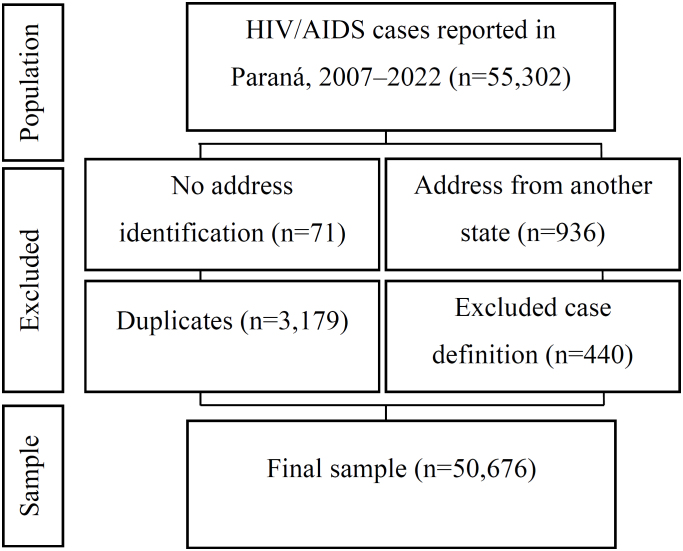
Population selection flowchart, exclusion criteria, and total sample of HIV/AIDS cases notified in SINAN from 2007 to 2022 in Paraná, Brazil.

To eliminate duplicates, a method similar to that used by the Ministry of Health was employed^12^. Duplications were identified by comparing the following fields: patient name, mother's name, and date of birth. Subsequently, the date of diagnosis and notification were considered. Records with the earliest diagnosis date were retained, and in case of a tie, the first notification date was considered. For duplicate records resulting from a new notification due to a change in the case definition criteria, meaning they had been previously reported as HIV infection and were later reported as AIDS, the same duplication exclusion method was applied, considering only one notification based on the hierarchy of diagnosis and notification date. A flowchart depicting the population selection process leading to the sample was created (Figure 1).

A time series was constructed for analysis according to the month of diagnosis. Incidence rates were calculated, with the numerator being the total number of HIV/AIDS cases diagnosed per month in the population aged 13 years or older residing in Paraná and the denominator being the total population of Paraná aged 13 years or older, based on population estimates for each year available in the DATASUS database – ("*Estudo de estimativas populacionais por município, sexo e idade 2000–2021*),"^13^ with multiplication by 100,000 inhabitants. It should be noted that the population estimate for the year 2021 is the most recent available based on age stratification.

For the temporal trend analysis, the Seasonal Decomposition of Time Series by LOESS (STL) method was applied^14^. Assuming an additive decomposition, the incidence rate of HIV/AIDS in month t (Yt) is given by the following formula: Y_(t=) S_(t) + T_t + R_t, where S_(t) represents the seasonal component, T_t is the trend component, and R_t is the residual or noise component. The Rstudio 3.5.2 software was used to construct the series and trend analysis graphs.

Subsequently, the Prais–Winsten method was employed to numerically estimate the temporal trend^15^. This method, based on linear regression, is given by the following formula: Yt = b0 + b1t + et, where b0 corresponds to a constant parameter, b1t corresponds to the slope of the line, and et is a random error. Through this linear regression, it is possible to estimate the value of the coefficient b1, applying a 95% confidence interval (CI) for this coefficient to calculate the monthly percentage change (MPC) and the 95% CI of the measure, as follows:^16^ MPC = [–1 + 10b1] * 100; 95% CI = [–1 + 10b1min.] * 100; [–1 + 10b1max.] * 100. When the rate is positive, the time series is considered increasing; when negative, it is decreasing; and when there is no significant difference between its value and zero^16^, it is considered stationary. For this analysis, the Stata 12.0 software (StataCorp LP, College Station, United States) was used.

For spatial analysis, HIV/AIDS rates were calculated for two periods: from 2007 to 2014 and from 2015 to 2022. The incidence rate was calculated for each municipality in the state of Paraná, with the numerator being the total number of HIV/AIDS cases diagnosed in the municipality during the respective period in the population aged 13 years or older, and the denominator being the total population of the municipality aged 13 years or older, based on population estimates for 2014 (for the first period) and for 2021 (for the second period), with multiplication by 100,000 inhabitants^13^.

To determine the presence or absence of a spatial pattern, rates were subjected to Moran's index, identifying clusters of areas with similar risk for the occurrence of the condition. A neighborhood matrix was constructed based on the binary contiguity criterion in Queen's case model^17^. The Moran's index ranges from –1 to +1, with values close to zero indicating the absence of spatial correlation and positive values indicating positive spatial autocorrelation. The results underwent pseudo-significance tests, involving 999 permutations to verify p-values for p<0.001^17^.

The Moran's I mirroring diagram was used for result interpretation. The diagram compares the values of a normalized variable with the average of the same variable for neighboring municipalities, where Q1 refers to positive variable values and positive neighbor averages; Q2 refers to negative variable values and negative neighbor averages; Q3 refers to positive variable values and negative neighbor averages; and Q4 refers to negative variable values and positive neighbor averages. Moran's I corresponds to the linear regression coefficient^18^.

To spatialize clusters, we utilized the digital cartographic database of the *Instituto Brasileiro de Geografia e Estatística* (IBGE)^19^. The maps of spatial clusters were generated by detecting regions with local spatial correlation (p<0.005), identified by BoxMap, with spatial statistical significance above 95%^17^. Critical areas were defined as those formed by municipalities falling into Q1 (high-high). For data processing and result mapping, the following software tools were used: GeoDa version 1.18.10 and QGIS version 3.16.7-Hannover.

The study was approved by the Ethics Committee of the State University of Londrina with Certificate of Ethical Consideration No. 00603718.6.0000.5231 and opinion No. 4.063.442 (June 2, 2020). Records were compiled securely, ensuring individual confidentiality and data security under the supervision of a single examiner.

## RESULTS

**Figure 2:**
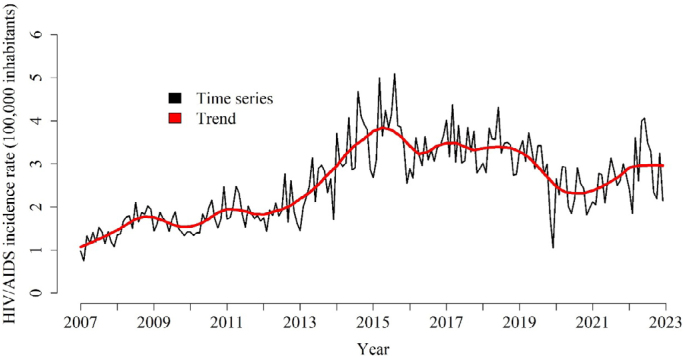
Temporal series and trend of HIV/AIDS incidence rates in Paraná, Brazil, from 2007 to 2022.

The temporal trend of HIV/AIDS incidence rates is depicted in Figure 2. From 2007 to 2014, the trend exhibited an upward trajectory, followed by a decline and subsequent plateau in 2016, which persisted until 2019. Subsequently, the trend showed another decline between 2020 and 2021 and began to exhibit a rising pattern toward the end of the study period.

According to the results of the Prais–Winsten regression for the period from 2007 to 2022, the monthly time series of HIV/AIDS incidence demonstrated a growth trend (MPC=2.14; 95%CI 1.16–3.13), signifying an average monthly increase of 2.14% for the series.

**Figure 3:**
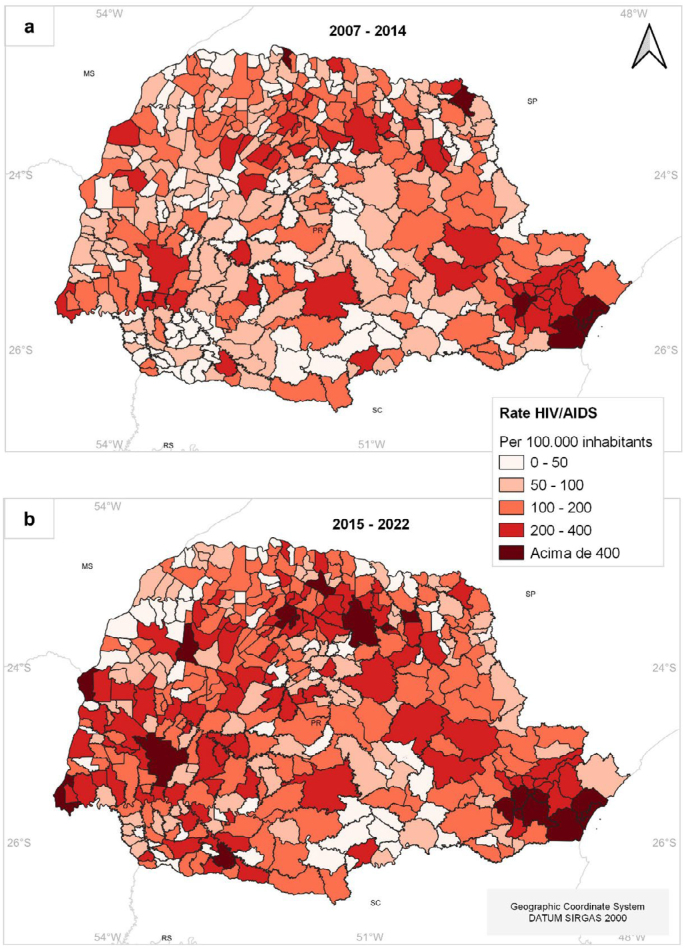
HIV/AIDS incidence rates in Paraná, Brazil, for the periods of 2007–2014 (a) and 2015–2022 (b).

Figure 3 presents the distribution and evolution of HIV/AIDS rates by municipalities in the state of Paraná, considering two analysis periods: 2007–2014 and 2015–2022.

For the first period (Figure 3a), the average number of cases was 105.64 per 100,000 inhabitants. It is noteworthy that 10% of municipalities in the state did not report any HIV/AIDS cases during this period. Higher rates were observed in municipalities in the state's Northern, Eastern, and Western regions, with concentrations of cases exceeding 400 per 100,000 inhabitants in the metropolitan region of Curitiba and the port city of Paranaguá. In terms of case quantity per 100,000 inhabitants, Paranaguá led the ranking during this period with 673.50 cases, followed by Pontal do Paraná with 556.45 cases, Matinhos with 531.91 cases, Guaratuba with 522.96 cases, and Pinhais with 487.12 cases.

For the second period (Figure 3b), the average number of cases registered was 159.20 per 100,000 inhabitants, with only 4% of municipalities reporting no cases. Paranaguá dropped to sixth place in the ranking, with 505.99 cases. The highest number of cases per 100,000 inhabitants was recorded in the municipalities of Guaratuba, with 654.56 cases, followed by Nova Fátima with 602.59 cases, Pinhais with 563.64 cases, Piraquara with 522.16 cases, and Curitiba with 518.26 cases.

It is noteworthy that, in both periods, the highest incidence rates are concentrated in municipalities in the metropolitan region of the capital and the coastal region, except for the second period, where Nova Fátima, located in the Northern Pioneer region of the state, stands out.

Regarding Moran's index, the results indicated the formation of significant clusters for both periods, with Moran's index values of 0.404 for the first period and 0.295 for the second period. Furthermore, the validation results (permutation test/z-value) suggest that the observed clusters are not random, indicating the presence of spatial autocorrelation with similar values among neighboring areas in terms of HIV/AIDS incidence rates, as shown in Table 1.

**Table 1:** Spatial autocorrelation of HIV/AIDS incidence, estimated by Moran's index and validated through permutation tests (Z-value) for the periods of 2007–2014 and 2015–2022 in Paraná, Brazil.

Periods	Moran's indexes	Z-values
2007–2014	0.404	13.1816
2015–2022	0.295	9.6780

**Figure 4:**
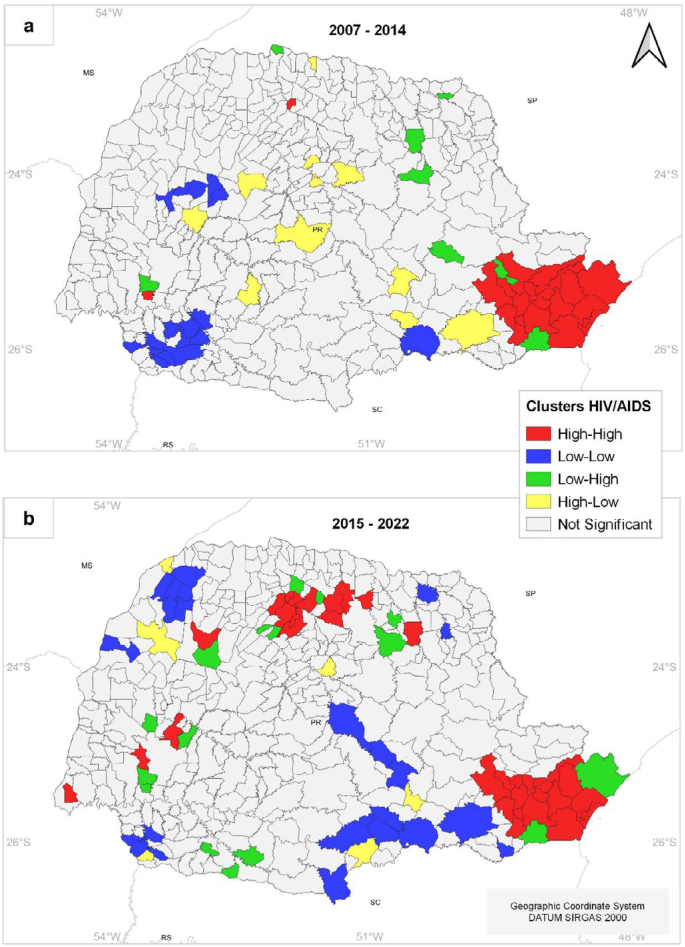
Spatial clusters of HIV/AIDS incidence rates in Paraná, Brazil, for the periods of 2007–2014 (a) and 2015–2022 (b).

For the first period (Figure 4a), a significant cluster (high-high) is observed in the metropolitan region of the state, encompassing 22 municipalities, including the capital, Curitiba, coastal municipalities like Matinhos, Guaraqueçaba, and Guaratuba, and the port city of Paranaguá. In the second period (Figure 4b), the cluster observed in the metropolitan region remains, and a new significant cluster (high-high) is observed further in the Northern Central region of the state, including the municipalities of Maringá, Rolândia, Sarandi, Cambé, Ângulo, Astorga, Arapongas, Ibiporã, Iguaraçu, Marialva, and Mandaguaçu.

## DISCUSSION

Throughout the 15-year analysis, a growing trend in HIV/AIDS incidence was observed in the state of Paraná. Higher incidence rates were concentrated in municipalities within the coastal and metropolitan regions of the capital. Subsequently, there was an increase in incidence rates in municipalities within the North Central and Western regions and bordering municipalities. Spatial clustering analysis identified significant clusters in the metropolitan region during the 1st and 2nd periods. Additionally, a new clustering was identified in the North Central region during the 2nd period, indicating the presence of geographical areas with a high concentration of HIV/AIDS incidence.

Although the temporal analysis revealed a monthly growth trend, signifying a consistent increase in incidence rates, fluctuations within the time series were identified among the years. These fluctuations can be attributed to changes and advancements in public policies and healthcare surveillance related to HIV/AIDS prevention, diagnosis, treatment, and monitoring^20^.

A pivotal moment was the Ministry of Health's decision to expand HIV diagnosis access through rapid testing (RT), decentralizing to primary healthcare (PHC) in 2012^21^. Following this decision, the temporal pattern shifted toward an upward trajectory, as evidenced by a significant increase of 132% in the distribution of RT in Paraná, rising from 315,375 in 2012 to 730,340 in 2015^22^. A similar increasing trend in 2013 was reported in a study conducted in the state of Paraná, suggesting that decentralization impacted the epidemic among young men who have sex with men (MSM)^23^.

The implementation of mandatory HIV notification in 2014^24^ may have directly influenced the peak of indicators observed in the same year. Diagnoses made before the change in criteria also began to be reported, resulting in increased incidence rates and a more accurate and comprehensive representation of the true extent of the epidemic in the state.

The stationary pattern in the time series from 2016 to 2019 may reflect the Ministry of Health's ongoing efforts to achieve the targets proposed by the Joint United Nations Programme on HIV/AIDS (UNAIDS) by 2020 through the 90-90-90 strategy: 90% of all people living with HIV know their status; 90% of diagnosed individuals receive antiretroviral therapy (ART); and 90% of those receiving treatment have suppressed viral loads (VL). Currently, the 95-95-95 targets are projected for 2030^25^. These findings suggest a strengthening of the healthcare system at the regional level^26^.

This strengthening is reflected in a study conducted in Paraná, where 93.1% of those diagnosed were on ART, and 90.0% had a VL below 50 copies of viral RNA/ml of blood^27^. Furthermore, the benefits of these achievements can be seen through multicenter studies. When HIV VL is suppressed, the risk of transmission in unprotected sexual relations, whether among MSM couples or heterosexuals, is effectively zero^28,29^. This underscores the importance of treatment adherence and consistent viral suppression.

The time series experienced a sharp decline between 2020 and 2021. It is important to note that this decline may be related to underreporting cases due to the coronavirus disease 2019 (COVID-19) pandemic.

The COVID-19 pandemic significantly impacted HIV-related healthcare services in various regions of the world. In KwaZulu-Natal, South Africa, there was a 48% decrease in HIV testing after the first national lockdown^30^. In the state of Paraná, reported cases to the Notifiable Diseases Information System (SINAN) decreased by 27% when comparing 2019 and 2020^4^.

Lockdowns and other restrictive measures adopted to contain the spread of COVID-19 negatively impacted HIV/AIDS programs^30^. Even during emergencies, it is crucial to maintain established plans to prevent setbacks in the progress made.

HIV/AIDS incidence maps in Paraná revealed that the epidemic was concentrated in the coastal and metropolitan regions, particularly in the metropolitan region of Curitiba, which houses 31.6% of the state's population and serves as a significant economic hub, attracting a workforce^31^. The high population density and extensive movement of people in the region contribute to the spread of infectious diseases.

This association is consistent with major urban centers, likely due to a higher transmission rate in densely populated areas, enabling the intensification of epidemic processes^32^. A study in Rio de Janeiro highlighted the association between urbanization and a high HIV incidence rate^33^. Geographical clustering is a common phenomenon in urban areas and has also been identified in other countries such as India and Ethiopia^34,35^.

In addition, larger cities often serve as primary healthcare providers for residents of smaller cities. In Paraná, they have a larger health infrastructure and serve as the headquarters of health regions (RS) such as Curitiba, Paranaguá, Londrina, Maringá, Cascavel, and Foz do Iguaçu^11^. A positive correlation between HIV/AIDS cases in cities serving as headquarters for macroregions was also found in a study by Mato Grosso do Sul^36^.

Notably, the four municipalities with the highest incidence rates in the first period of analysis in Paraná were located in the coastal region. The presence of spatial autocorrelation in this region reinforces these findings, as significant clusters indicate that geographically close areas tend to have similar incidence patterns.

The economic, demographic, and social dynamics of the coastal region are influenced by the dynamism of the Port of Paranaguá, and other factors such as the location of the state's major roadways, high fluctuating population density, mobility, seasonal labor migration, and tourism are elements that can contribute to the propagation and maintenance of HIV^34,37^. These characteristics, also recognized in border regions, represent critical points facilitating multiple casual partners, unprotected sexual relations, drug concentration, and sexual exploitation^38^. This implies implementing prevention strategies tailored to the local context, taking into account the population-specific characteristics within the context of combined HIV prevention^39^.

The significant clusters in the North Central region in recent years, particularly around Maringá and its adjacent municipalities, as well as municipalities in the metropolitan region of Londrina, may be related to constant population growth, geoeconomic factors, strong education, healthcare, and transportation infrastructures, and migratory patterns^40^.

Migration has played a significant role in population growth, especially in Maringá^41^. The expansion of higher education institutions in the last decade, attracting prospective students and academics, has contributed to changes in urban and interurban space, generating employment and, consequently, socio-spatial impacts^42^.

Besides, it is known that migration and education are considered important social determinants in the HIV epidemic dynamics^43,44^. Changes in the social environment, increased mobility, and exposure to different social networks can lead to risky sexual behaviors such as multiple sexual partners or inconsistent condom use^37,44^. Despite good knowledge of prevention among the university population, risky behaviors continue, which are often associated with alcohol and drug use^45^. In China, the proportion of cases among individuals with a university degree or higher has substantially increased, and the number of university students diagnosed with AIDS grows by 30–50% annually^46,47^.

In our findings, spatial clusters with a high risk for HIV/AIDS were found in the municipalities of Curitiba and Maringá, with the highest human development index (HDI), higher levels of education among the population, and a lower proportion of individuals without income^9^. The positive relationship between the high risk of HIV/AIDS in areas with high economic activity, higher socioeconomic status, and better indicators measuring population living conditions was evident in other regions of Brazil and around the world^33,36,48-50^. Nevertheless, it is essential to consider the social context and living conditions in each geographical area, as different territories may be more affected by socioeconomic factors regarding the incidence of the disease^2,33,36^.

The absence of reported HIV/AIDS cases in some cities, particularly smaller ones in rural areas, requires attention from municipal and state healthcare management. These areas are recognized as having a higher chance of underreporting^51^. Measures such as promoting rapid testing in primary care clinics, encouraging spontaneous visits, showing sensitivity to vulnerable populations, and establishing strong patient-provider relationships can contribute to changing this epidemiological landscape^52^. These strategies should be prioritized not only in cities that have not reported cases but also in regions that have shown significant spatial clustering of low incidence (low-low) such as the extreme southwestern, southeastern, and northwestern regions.

Our analysis had a large sample and broad geographical coverage across Paraná. However, the study has some limitations. First, the study was conducted based on data available in SINAN, which may not reflect exact numbers due to potential underreporting and underdetection of cases. Second, incidence rates were calculated based on population estimates, which may not fully reflect the actual population size during the period.

It is hoped that the results of this study will contribute to improving healthcare actions in the state of Paraná across various contexts, providing insights for developing strategies to achieve better future outcomes in epidemiological indicators.
